# Heterologous expression in *Toxoplasma gondii* reveals a topogenic signal anchor in a *Plasmodium* apicoplast protein

**DOI:** 10.1002/2211-5463.12527

**Published:** 2018-10-22

**Authors:** Aishwarya Narayan, Pragati Mastud, Vandana Thakur, Pradipsinh K. Rathod, Asif Mohmmed, Swati Patankar

**Affiliations:** ^1^ Department of Biosciences and Bioengineering IIT Bombay Mumbai India; ^2^ International Centre for Genetic Engineering and Biotechnology New Delhi India; ^3^ Department of Chemistry University of Washington Seattle WA USA

**Keywords:** apicoplast, *Plasmodium*, signal anchor, split‐GFP, *Toxoplasma*, transmembrane

## Abstract

Glutathione peroxidase‐like thioredoxin peroxidase (PfTPx_Gl_) is an antioxidant enzyme trafficked to the apicoplast, a secondary endosymbiotic organelle, in *Plasmodium falciparum*. Apicoplast trafficking signals usually consist of N‐terminal signal and transit peptides, but the trafficking signal of PfTPx_Gl_ appears to exhibit important differences. As transfection is a protracted process in *P. falciparum*, we expressed the N terminus of PfTPx_Gl_ as a GFP fusion protein in a related apicomplexan, *Toxoplasma gondii*, in order to dissect its trafficking signals. We show that PfTPx_Gl_ possesses an N‐terminal signal anchor that takes the protein to the endoplasmic reticulum in *Toxoplasma*—this is the first step in the apicoplast targeting pathway. We dissected the residues important for endomembrane system uptake, membrane anchorage, orientation, spacing, and cleavage. Protease protection assays and fluorescence complementation revealed that the C terminus of the protein lies in the ER lumen, a topology that is proposed to be retained in the apicoplast. Additionally, we examined one mutant, responsible for altered PfTPx_Gl_ targeting in *Toxoplasma*, in *Plasmodium*. This study has demonstrated that PfTPx_Gl_ belongs to an emergent class of proteins that possess signal anchors, unlike the canonical bipartite targeting signals employed for the trafficking of luminal apicoplast proteins. This work adds to the mounting evidence that the signals involved in the targeting of apicoplast membrane proteins may not be as straightforward as those of luminal proteins, and also highlights the usefulness of *T. gondii* as a heterologous system in certain aspects of this study, such as reducing screening time and facilitating the verification of membrane topology.

**Abbreviations**
ACP: acyl carrier protein
BiP: binding immunoglobulin protein
ERAD: ER‐associated degradation system
ER: endoplasmic reticulum
HSP70: heat shock protein
PDI: protein disulfide isomerase
PfiTPT: *Plasmodium falciparum* apicoplast innermost membrane phosphate translocator
PfoTPT: *Plasmodium falciparum* apicoplast outermost membrane phosphate translocator
PfTPx_Gl_: *Plasmodium falciparum* glutathione peroxidase‐like thioredoxin peroxidase
SELMA: symbiont‐derived ERAD‐like machinery
TgTPx: *Toxoplasma gondii* thioredoxin peroxidase
TIC: translocon of the inner membrane of the chloroplast
TOC: translocon of the outer membrane of the chloroplast

The apicoplast is a secondary endosymbiotic organelle, bound by four membranes, found in most members of the phylum *Apicomplexa* 1. Targeting proteins into and across these multiple membranes is a specialized task that invokes interesting adaptations. For proteins trafficked to the lumen of the apicoplast, a bipartite targeting sequence is employed. A signal peptide that enables co‐translational uptake at the ER is cleaved in the ER lumen, thereby exposing the transit peptide. When this preprotein reaches the apicoplast by the endomembrane system, the exposed transit peptide facilitates uptake across its inner membranes by translocons. Transit peptide cleavage then releases the mature protein into the apicoplast lumen 2.

Due to the absence of an apicoplast in the human host of the malaria parasite *Plasmodium falciparum*, this specialized targeting mechanism may be exploited in the development of antimalarials. Once the outermost apicoplast membrane, contiguous with the endomembrane system, is traversed, the protein is transported across the other three membranes by translocons that bear homology to the ERAD system (called the symbiont‐derived ERAD‐like machinery, or SELMA, in complex plastids) 3, 4, 5 and to the TOC and TIC complexes (translocons of the outer and inner membranes of the chloroplast, named similarly in complex plastids) 6 in the periplastid, outer, and inner apicoplast membranes, respectively. Chaperones such as BiP (binding immunoglobulin protein), PDI (protein disulfide isomerase), and HSP70 (heat shock protein) are also involved in these membrane translocations. Given that over 500 nuclear‐encoded proteins are targeted to the apicoplast, all of these proteins involved in apicoplast protein trafficking can serve as important drug targets that are capable of disrupting parasite viability when inhibited 7.

In trafficking from the ER to the apicoplast, the scientific community still debates the role of vesicles and the Golgi 8, 9, 10, 11. There also appear to be differences in the trafficking of apicoplast luminal proteins when compared with those trafficked to the membranes. Indeed, a recent paper indicates that the apicoplast outer membrane phosphate translocator of *P. falciparum* (PfoTPT) is devoid of a canonical bipartite targeting sequence and instead uses a signal anchor to localize to the endomembrane system 12.

Another apicoplast protein, glutathione peroxidase‐like thioredoxin peroxidase (PfTPx_Gl_, PlasmoDB Gene ID PF3D7_1212000) targets to the apicoplast via the Golgi, unlike the apicoplast luminal acyl carrier protein (ACP), that in the same paper was found to route independently of the Golgi 2. Additionally, in another report, PfTPx_Gl_ trafficking is inhibited by treatment with tetrafluoroaluminate, which blocks the fusion of heterotrimeric G protein‐dependent vesicles to their target membranes, while ACP trafficking is completely unaffected 13. Incidentally, the bipartite targeting signal of ACP is cleaved during this process, while the size of PfTPx_Gl_ remains unaltered 8, 10. Further investigation revealed that PfTPx_Gl_ is anchored to the apicoplast membrane 13. This led us to speculate that membrane anchorage itself might play a role in the altered targeting pathway of this protein when compared with that of luminal ACP.

Kehr *et al*. 14 have shown that the N‐terminal 47 residues of PfTPx_Gl_ target GFP to the apicoplast and the cytosol in *Plasmodium*. While this work confirmed the adequacy of the N terminus for apicoplast targeting, a deeper understanding of the signals within this region that mediate each step of the pathway requires extensive mutational analysis, a procedure that takes months due to the low efficiencies of transfection in *P. falciparum* 15, 16. In contrast, *Toxoplasma gondii*, an apicomplexan closely related to *P. falciparum*, is easier to transfect, with the efficiency of transfection being orders of magnitude higher than in *Plasmodium*. Consequently, transient transfectants may be studied within hours, while parasites that have integrated foreign DNA under drug pressure revive in a matter of days 17.

Apart from technical benefits, there is another rationale for using *T. gondii* to study the PfTPx_Gl_ protein. The ER–Golgi secretory route is broadly conserved across species, more so for closely related apicomplexans such as *P. falciparum* and *T. gondii*. It has been experimentally demonstrated that trafficking to the apicoplast is a branch of the secretory route 2. In keeping with this theme, the N‐terminal apicoplast targeting signal of *Toxoplasma* ACP has successfully targeted GFP to the *Plasmodium* apicoplast 18. Similarly, the N termini of *Plasmodium* proteins PfiTPT and DOXP reductoisomerase also targeted GFP to the *Toxoplasma* apicoplast 9, 19. Such examples demonstrate that apicoplast signals are conserved, at least for certain proteins, between these organisms.

The factors listed above endorse *Toxoplasma* as a suitable preliminary system for the rapid screening of sequence determinants in *Plasmodium* proteins, such as PfTPx_Gl_, that contribute to biological phenomena that are similar between the two parasites. Therefore, the N‐terminal 47 residues of PfTPx_Gl_ were codon optimized for expression as a GFP fusion protein in *Toxoplasma* and used to dissect the key sequence features of this region.

## Materials and methods

### Ethics approval and consent to participate

Human blood for *Plasmodium* culture was collected from a blood bank. The donors gave their written informed consent. All procedures conformed to the standards set by the Declaration of Helsinki.

#### Plasmid construction

To generate the plasmid expressing PfTPx_Gl_47‐EGFP, codon optimized tandem oligonucleotides, encoding residues 1–47 of PfTPx_Gl_ and a triple glycine linker, were inserted upstream of EGFP in the vector pCTG‐EGFP 20. As this protein has multiple in‐frame methionines, the Kozak context of these methionines was retained so as to match the relative Kozak frequencies between them in *Plasmodium* 21, 22. The plasmid expressing P30‐mCherry‐HDEL was generated by replacing YFP‐HDEL in the ptub‐P30‐YFP‐HDEL vector 23 with mCherry‐HDEL amplified from ptub‐mCherry. Plasmids encoding PfTPx_Gl_S‐EGFP and PfTPx_Gl_ΔS‐EGFP were generated by replacing the PfTPx_Gl_47 region in pCTG‐PfTPx_Gl_47‐EGFP with PfTPx_Gl_S or PfTPx_Gl_ΔS. In the PfTPx_Gl_ΔS‐EGFP mutant, a methionine was included at the translation start site. The plasmid encoding ERS1–10 was generated by replacing YFP‐HDEL in ptub‐P30‐YFP‐HDEL with GFPS1–10 amplified from the pCMV‐mGFP 1‐10 Hyg Amp (Sandia Biotech, Albuquerque, NM, USA) including a C‐terminal ER retention sequence (HDEL). The plasmid encoding CyS1–10 was generated by replacing EGFP in pCTG‐EGFP with GFPS1–10. The plasmid encoding PfTPx_Gl_47‐S11 was generated by replacing EGFP in the construct expressing PfTPx_Gl_47‐EGFP with GFPS11 amplified from pCMV‐mGFP Cterm S11 Neo Kan (Sandia Biotech). Plasmids encoding the PfTPx_Gl_ fusion proteins with ACP [ACP_SP_‐PfTPx_Gl_ΔS‐EGFP and PfTPx_Gl_S‐ACP_TP_‐EGFP] were generated by overlap extension PCR with vectors pCTG‐PfTPx_Gl_47‐EGFP and pCTG‐ACP‐HA using Phusion polymerase (NEB) 24. The plasmid encoding PfTPx_Gl_47‐M26A‐EGFP was synthesized using a mismatched forward primer to amplify pCTG‐PfTPx_Gl_47‐EGFP with KAPA HiFi DNA polymerase.

To generate the plasmids expressing GFP fusion proteins in *Plasmodium*, the region encoding the first 47 residues of PfTPx_Gl_, including the start codon context of 12 bp and a triple glycine linker, was amplified from the pET28a‐PfTPx_Gl_ plasmid containing PfTPx_Gl_ cloned from *Plasmodium* mRNA in the case of PfTPx_Gl_47‐GFP, and synthesized as tandem oligonucleotides in the case of PfTPx_Gl_47‐M26A‐GFP. Each of these fragments was inserted upstream of GFP in the pSSPF2 vector 25. Primers used are listed in Table 1.

**Table 1:** Primers used in plasmid construction. Restriction sites are underlined and the mismatch regions are italicized. All primer sequences are shown from 5ʹ to 3ʹ

Construct	Forward primer	Reverse primer
PfTPx_Gl_47‐EGFP (oligonucleotides)	F1:CTAGCAACATGTTCTTCTCAATGTTCATCAAGTTCATCTTGCCGATCTCTTTCATCTGCTACAACTTCGG	R1:GAACATGTTGAACTTCTTGCCGAAGTTGTAGCAGATGAAAGAGATCGGCAAGATGAACTTGATGAACATTGAGAAGAACATGTTG
	F2:CAAGAAGTTCAACATGTTCTCTTACTTCCAGAAGATCAAGGTCTCTGAGCAGGAGTTGTTGTCTTCTATCTACGACTACGGCGGCGGCC	R2:CTAGGGCCGCCGCCGTAGTCGTAGATAGAAGACAACAACTCCTGCTCAGAGACCTTGATCTTCTGGAAGTAAGA
P30‐mCherry‐HDEL	GAACCTAGGGTGAGCAAGGGCGAGGAG	CATTCTTAAGCTACAACTCGTCGTGCTTGTACAGCTCGTCCATG
PfTPx_Gl_S‐EGFP	GATCTGCTAGCAACATGTTCTTCTCAATGTTCATC	CGCCCTAGGGCCGCCGCCGCCGAAGTTGTAGCA
PfTPx_Gl_ΔS‐EGFP	GATCTGCTAGCAACATGAAGAAGTTCAACATGTTC	CGCCCTAGGGCCGCCGCCGTAGTCGTAG
PfTPx_Gl_47‐S11	GAACCTAGGGGCGACGGCGGCAGCGGC	CGTTCTGCAGTTTATGTGATGCCGGCGGC
ERS1–10	GAACCTAGGGCGGTTTCGAAAGGCGAGGAG	CGTTCTTAAGCTACAACTCGTCGTGTTTCTCGTTTGGGTCTTTG
CyS1–10	GAAGCTAGCAAAATGGTTTCGAAAGGCGAG	CGTTCTGCAGGTTATTTCTCGTTTGGGTC
ACP_SP_‐PfTPx_Gl_ΔS‐EGFP	CATTTTTTCTTGAATTCCCTTTTAGATCTGCTAGCAACATGGAGATGCATCCCCG	CTTGATCTTCTGGAAGTAAGAGAACATGTTGAACTTCTTACCGTAGGAAGAAGCAATGG
PfTPx_Gl_S‐ACP_TP_‐EGFP	GATCTCTTTCATCTGCTACAACTTCGGCTTTGTGTCACCAGGCCTGATC	GCTCACCGCCCTAGGGCCGCCGCCATCAGAACTCGCCTCGTCGG
PfTPx_Gl_47‐M26A‐EGFP	AAGAAGTTCAAC*GC*GTTCTCTTACTTC	GCCGAAGTTGTAGCAGATGAAAGAG
PfTPx_Gl_47‐GFP (Pf)	GGAAGATCTAATATAAAAAATATGTTCTTCTCAATGTTTATTAAATTC	CGCCCTAGGTCCTCCTCCATAATCATAAATGGATG
PfTPx_Gl_47‐M26A‐GFP (Pf)	F1:GATCTAATATAAAAAATATGTTCTTCTCAATGTTTATTAAATTCATTTTACCTATATCTTTTATATGCTACAAT	R1:ATTAAATTTCTTTCCAAAATTGTAGCATATAAAAGATATAGGTAAAATGAATTTAATAAACATTGAGAAGAACATATTTTTTATATTA
	F2:TTTGGAAAGAAATTTAAT*GCA*TTTAGTTATTTTCAAAAAATCAAAGTATCAGAACAGGAATTATTATCATCCATTTATGATTATGGAGGAGGA C	R2:CTAGGTCCTCCTCCATAATCATAAATGGATGATAATAATTCCTGTTCTGATACTTTGATTTTTTGAAAATAACTAAA*TGC*

#### Parasite culture and transfection

Methods used for *T. gondii* RH strain maintenance, harvest, transfection, and selection of stable and clonal transfectants were as described previously 17 with minor modifications. For transfections, tachyzoites were electroporated at 1.5 kV/50 Ω/25 μF. Following this, each chamber slide was inoculated with 10^7^ parasites and fixed after 24 h to observe transient transfectants. Stable lines were generated by restriction enzyme‐mediated integration using NotI and selecting for growth in 20 μm chloramphenicol.

*Plasmodium falciparum* strain 3D7 was cultured with 4% hematocrit in RPMI media (Invitrogen, Carlsbad, CA, USA) supplemented with 0.5% albumax using a published protocol 26. Parasite cultures were synchronized by two consecutive sorbitol treatments, as described by Lambros and Vanderberg 27, 4 h apart. Synchronized *P. falciparum* 3D7 ring stage parasites were transfected with 100 μg of purified plasmid DNA (Plasmid Maxi Kit, Qiagen, Valencia, CA, USA) by electroporation (310 V, 950 μF) 28. The transfected parasites were selected for growth in 2.5 nm blasticidine.

#### Microscopy

*Toxoplasma* tachyzoites were prepared for imaging as described previously 17 with minor modifications. Parasites were incubated in primary antibody (1 : 200 polyclonal rabbit anti‐GFP, Invitrogen) for 4 h and in secondary antibody (1 : 400 anti‐rabbit Alexa 568) for 1.5 h. Slides were stored at 4 °C and imaged in under 48 h postfixation. Imaging was carried out with the Zeiss LSM 780 NLO confocal microscope (Plan‐Apochromat 100X/1.4 NA). Each planar section was averaged eight times. Maximum intensity projections were generated from the Z‐stacks. Images were processed using Fiji (imagej) 29, with only linear adjustments to brightness and contrast applied consistently across all pixels in an image. No nonlinear adjustments, such as changes to the gamma settings or curves, were implemented. Images were processed minimally, only to the extent of reducing background fluorescence external to the parasite, while ensuring that no other features were altered. For each of the transfectants imaged in this study, a minimum of 200 parasites were observed microscopically to ensure consistency in morphology, of which the images of approximately 10 rosettes were captured for the purposes of documentation.

*Plasmodium falciparum* transgenic parasite lines were prepared for indirect immunofluorescence assays as described previously 30. Parasites were incubated in rabbit anti‐PfClpP (1 : 200) 31 and mouse anti‐GFP (1 : 500, Roche, Basel, Switzerland) primary antibodies diluted in 3% BSA (bovine serum albumin), 1× PBS (phosphate‐buffered saline; 137 mm NaCl, 2.7 mm KCl, 10 mm Na_2_HPO_4_, 2 mm KH_2_PO_4_, pH 7.4). Subsequently, following incubation in the secondary antibodies goat anti‐rabbit Alexa 594 (1 : 250, Sigma‐Aldrich, St. Louis, MO, USA) and goat anti‐mouse Alexa 488 (1 : 250), parasite nuclei were stained with DAPI (2 μg·mL^−1^) for 30 min at 37 °C. The immunostained parasites were viewed using a Nikon A1 confocal laser scanning microscope. Colocalization with the apicoplast marker was verified using methods described previously 32. Three‐dimensional reconstructions of the parasites were generated using the imaris 7.01 software by Bitplane (Belfast, Northern Ireland, UK). To assess the extent of colocalization with the apicoplast marker PfClpP, the Pearson's correlation coefficient was calculated for each parasite by using the Nikon NIS‐Elements colocalization software. A dot plot of the Pearson's correlation coefficient for colocalization with the apicoplast marker was plotted for parasite lines expressing PfTPx_Gl_47‐GFP and PfTPx_Gl_47‐M26A‐GFP using the graphpad prism 7 software (La Jolla, CA, USA).

#### Sample preparation for SDS/PAGE and western blotting

Tachyzoites were harvested as described previously 17 and counted using a hemocytometer. Following this, the parasites were subjected to any necessary extractions as described in Sections 2.5 and 2.6. For whole parasite lysates, the tachyzoites were resuspended in an appropriate volume of PBS. An equal volume of 2X protein loading sample buffer with dithothreitol [100 mm Tris/Cl (pH 6.8), 4% (w/v) SDS, 0.2% (w/v) bromophenol blue, 20% (v/v) glycerol, 200 mm dithiothreitol] was added to the resuspended tachyzoites. The sample was then boiled for 3 minutes at 100 °C before loading on the gel. For western blotting, a PVDF membrane [Millipore Immobilon‐P Transfer Membrane (IPVH00010, 0.45 μm)] was activated in methanol for 5 minutes, following which both the gel and the membrane were equilibrated in transfer buffer (25 mm Tris, 192 mm glycine, 20% methanol, pH 8.3). Western blotting by wet transfer was then carried out in a Genetix GX‐ZY5 blotting apparatus at 4 °C, either overnight (minimum 12 h) at 40 mA, or for 2 h at 250 mA, with the instrument packed in ice to prevent overheating. Following protein transfer, the PVDF membrane was blocked in 3% BSA‐PBS for 1 h, incubated in primary antibodies [1 : 500 mouse anti‐GFP (Roche)] for 4 h, and incubated in HRP‐conjugated secondary antibodies for 1.5 h prior to development with diaminobenzidine.

#### Membrane protein extraction

Extracellular parasites (10^8^) were hypotonically lysed in 1 mL of sterile deionized water containing protease inhibitors (Sigma) by six freeze–thaw cycles (5 min each/liquid nitrogen/37 °C). After concentrating over ten times using centrifugal filter units (Amicon ultra‐3K, Merck, Kenilworth, NJ, USA), samples were centrifuged (30 000 ***g***/30 min/4 °C) 33. The supernatant, containing all soluble proteins, constituted the soluble fraction. The pellet was lysed in 1% SDS (1 h/room temperature) to extract all membrane proteins and centrifuged (30 000 ***g***/30 min/4 °C); 30 μL each of the soluble and pellet (1% SDS supernatant) fractions was resolved by SDS/PAGE and subjected to western blotting as described previously. Here, the PVDF membrane was blocked in 3% BSA‐PBS for 1 h, incubated in primary antibodies [1 : 500 mouse anti‐GFP (Roche) and 1 : 10 000 rabbit anti‐BiP (a kind gift from J. Bangs) 34 for 4 h, and incubated in HRP‐conjugated secondary antibodies for 1.5 h prior to development with diaminobenzidine.

#### Differential permeabilization of membranes

All parasites in this assay were freshly harvested to ensure the integrity of all membranes prior to treatment. Extracellular tachyzoites (6 × 10^7^) resuspended in assay buffer (250 mm sucrose, 20 mm Tris/Cl, 0.5 mm CaCl_2_, pH 7.4) were aliquoted into six tubes of 95 μL each. Following a 30‐minute incubation on ice in digitonin (0.005%), Triton X‐100 (1%), and/or EDTA (10 mm) as required, samples were treated with 500 μg·mL^−1^ thermolysin (5 minutes/37 °C). 10 mm EDTA was then added to the samples to stop the reaction. After lysis in 1% SDS, samples were precipitated by the methanol–chloroform method and subjected to western blotting as described previously.

## Results

Immunofluorescence data indicate that glutathione peroxidase‐like thioredoxin peroxidase (PfTPx_Gl_) is trafficked not just to the apicoplast, but also to the mitochondrion and the cytosol in *P. falciparum* 10, 14. Small molecule inhibitors have previously been used for the characterization of trafficking pathways used by PfTPx_Gl_ to reach the apicoplast and mitochondrion. Inhibition of vesicle fusion by blocking heterotrimeric G proteins showed a complete disruption of PfTPx_Gl_ targeting to the apicoplast and only a partial disruption of targeting to the mitochondrion 13. This suggests that further experiments are needed to understand the mitochondrial trafficking of PfTPx_Gl_. Therefore, this paper is primarily focused on apicoplast targeting alone.

Notably, the homologue of PfTPx_Gl_ in *T. gondii* is an alternatively spliced thioredoxin peroxidase (TgTPx). While the shorter splice variant (TgTPx1/1) is localized to the cytosol, the other (TgTPx1/2) is dually targeted to the apicoplast and the mitochondrion 35. This is very similar to the targeting observed for native PfTPx_Gl_ in *Plasmodium* when analyzed by immunofluorescence 10.

Published literature documents the targeting of a C‐terminal GFP fusion of the first 47 residues of the antioxidant enzyme PfTPx_Gl_ to the apicoplast and the cytosol in *Plasmodium* 14. The protein is predicted to have an N‐terminal signal peptide; however, no clear transit peptide is observed by bioinformatics. Therefore, to study the signals that direct PfTPx_Gl_ to the ER, Golgi, and eventually the apicoplast, the first 47 residues of PfTPx_Gl_ were expressed as PfTPx_Gl_47‐EGFP in *T. gondii* and dissected by mutational analysis.

### The N terminus of PfTPx_Gl_ possesses a signal sequence

For expression in *T. gondii*, the N‐terminal 47 residues of PfTPx_Gl_ were codon optimized and expressed as a C‐terminal EGFP fusion protein, PfTPx_Gl_47‐EGFP, in the RH strain. As this protein has multiple in‐frame methionines, the Kozak context of these methionines was retained so as to match the relative Kozak frequencies between these methionines in *P. falciparum* 21, 22. Experiments were performed using clonal lines generated under drug pressure.

**Figure 1:**
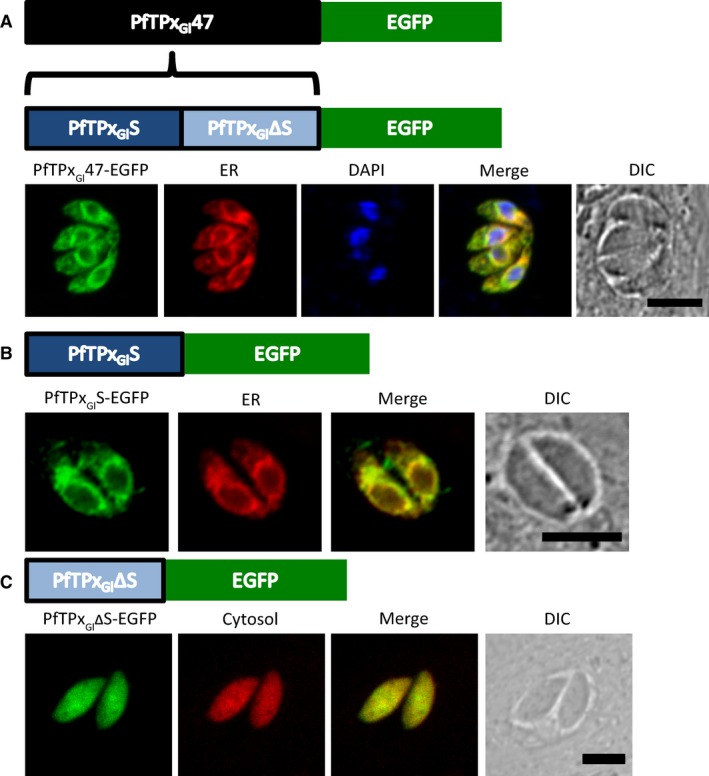
The PfTPx_Gl_47‐EGFP localization in *Toxoplasma gondii*. Microscopic images of parasites expressing (A) PfTPx_Gl_47‐EGFP (green), (B) PfTPx_Gl_S‐EGFP (green), in which the residues 1–21 of PfTPx_Gl_ are fused to EGFP, and (C) PfTPx_Gl_ΔS‐EGFP (green), with a methionine residue followed by residues 22–47 fused to EGFP. ER marker protein P30‐mCherry‐HDEL (red) and cytosolic protein Hexokinase‐mCherry (red) have been used to label the respective compartments. Scale bar = 5 μm.

In these lines, PfTPx_Gl_47‐EGFP was observed to target to the ER in every single parasite, as confirmed by colocalization with the ER marker P30‐mCherry‐HDEL (Fig. 1A) in a distinctly perinuclear structure characteristic of the ER. Notably, what appeared to be mitochondrial and apicoplast localization was observed in parasites transiently transfected with this construct (data not shown). However, we chose not to pursue these phenotypes as they were not robust and were observed only in very few of several hundred parasites screened.

It is known that entry into the ER is the first step in trafficking to the apicoplast. Additionally, PfTPx_Gl_ is known to route via the ER and the Golgi in *Plasmodium* 10. This motivated us to exploit the ER localization of this protein to understand the signals involved in this first step of organellar targeting by mutational analysis.

Targeting to the ER requires a signal sequence, the length and location of which was predicted using SignalP 3.0 36 (suitable for apicomplexans 37) and the Phobius server 38. The predicted signal peptide was between residues 1–21. A fusion of residues 1–21 with EGFP was expressed in parasites as PfTPx_Gl_S‐EGFP. Interestingly, native EGFP fluorescence was absent in these parasites and anti‐GFP antibodies were used to confirm localization, suggesting that EGFP was misfolded. As seen in Fig. 1B, PfTPx_Gl_S‐EGFP colocalized with the ER marker, P30‐mCherry‐HDEL. This led us to conclude that residues 1–21 were indeed sufficient for ER targeting.

Residues 22–47, when expressed with an N‐terminal methionine and C‐terminal EGFP tag as PfTPx_Gl_ΔS‐EGFP, did not target to the ER and instead remained in the cytosol (Fig. 1C), as confirmed by colocalization with the cytosolic marker Hexokinase‐mCherry (a kind gift from D. Shanmugam) 39. This implied that the sequence was unable to facilitate uptake at the ER or at either of the endosymbiotic organelles. Notably, the protein PfTPx_Gl_ΔS‐EGFP passively diffused across the nuclear pore complex owing to its small size (<40 kDa) 40.

### PfTPx_Gl_ is anchored to the membrane by an N‐terminal transmembrane domain

**Figure 2:**
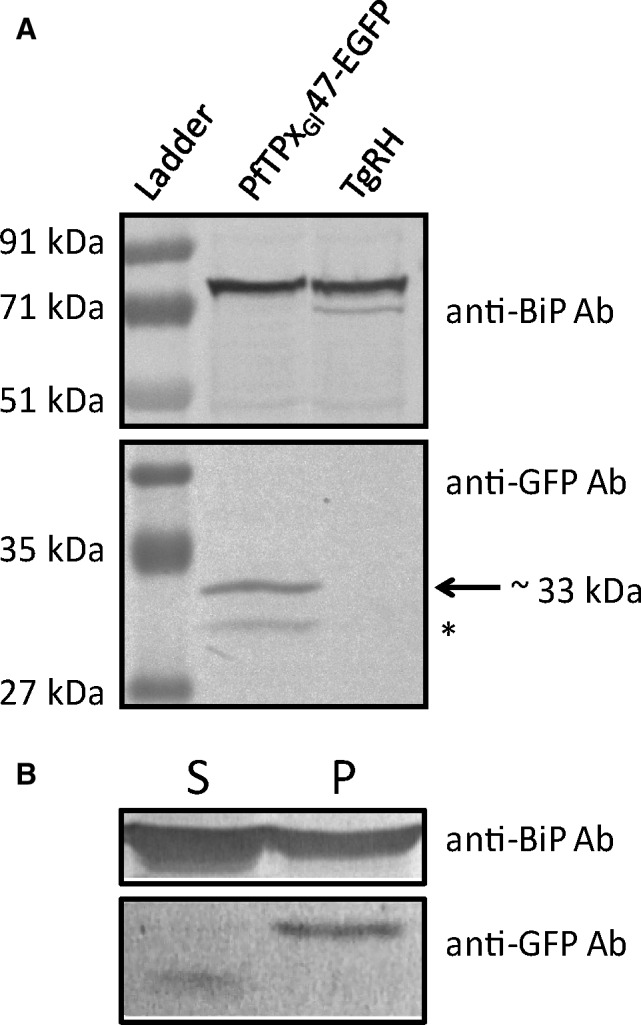
Western blots of parasites expressing PfTPx_Gl_47‐EGFP. Western blot analyses for (A) whole parasite lysates of PfTPx_Gl_47‐EGFP transfectants and untransfected *Toxoplasma gondii* RH parasites using anti‐GFP and anti‐BiP antibodies (B) fractionation of PfTPx_Gl_47‐EGFP parasites into soluble [S] and membrane/pellet [P] fractions. The input for the fractionation experiments gave results identical to those of the whole parasite lysate (A).

Targeting to the ER lumen typically involves cleavage of the signal peptide. The cleavage site itself is determined by the (−3, −1) rule 41, where small, neutral residues at positions −3 and −1 from a site increase the propensity of cleavage at that site. Western blotting of parasites expressing PfTPx_Gl_47‐EGFP revealed that the band was at the size predicted in the absence of cleavage, ~33 kDa (Fig. 2A). No band was evident in the control lane, which contained total lysates of untransfected parasites of the RH strain. A faint band was visible below the 33 kDa PfTPx_Gl_47‐EGFP band, either a product of GFP degradation that has been reported previously in the literature 18, partial cleavage at residues 21–22, or alternative translation initiation (Met^26^). Even in light of these other possibilities, a majority of the protein still remains in the unprocessed state, at the full size of ~33 kDa. The lack of processing could be indicative of a signal anchor as proposed elsewhere 12, 42. Additionally, both 1–47 and 1–21 residue EGFP fusion proteins remain in the ER despite the absence of identifiable ER retention signals. It has been suggested that transmembrane domains have the potential to retain proteins in specific compartments 43. Taken together, these observations led us to test whether the protein was anchored to the membrane by an N‐terminal transmembrane domain. Indeed, PfTPx_Gl_ has been demonstrated to be membrane bound in *P. falciparum* 13.

Multiple freeze–thaw cycles in a hypotonic solution separated the soluble contents from the membrane fraction. Western blots revealed that the ER luminal control protein BiP (binding immunoglobulin protein) partitioned primarily into the soluble fraction (Fig. 2B) 23. Incomplete partitioning of luminal proteins is a common occurrence documented in other reports 4. Interestingly, full‐length PfTPx_Gl_47‐EGFP partitioned entirely into the membrane fraction, while the shorter protein partitioned into the soluble fraction.

### The C‐terminal domain of PfTPx_Gl_ lies in the ER lumen

It has been suggested that the orientation acquired by a membrane protein at the ER is retained at its final destination 12, 42. Therefore, understanding the orientation of PfTPx_Gl_ in the ER might reveal its orientation in the apicoplast.

**Figure 3:**
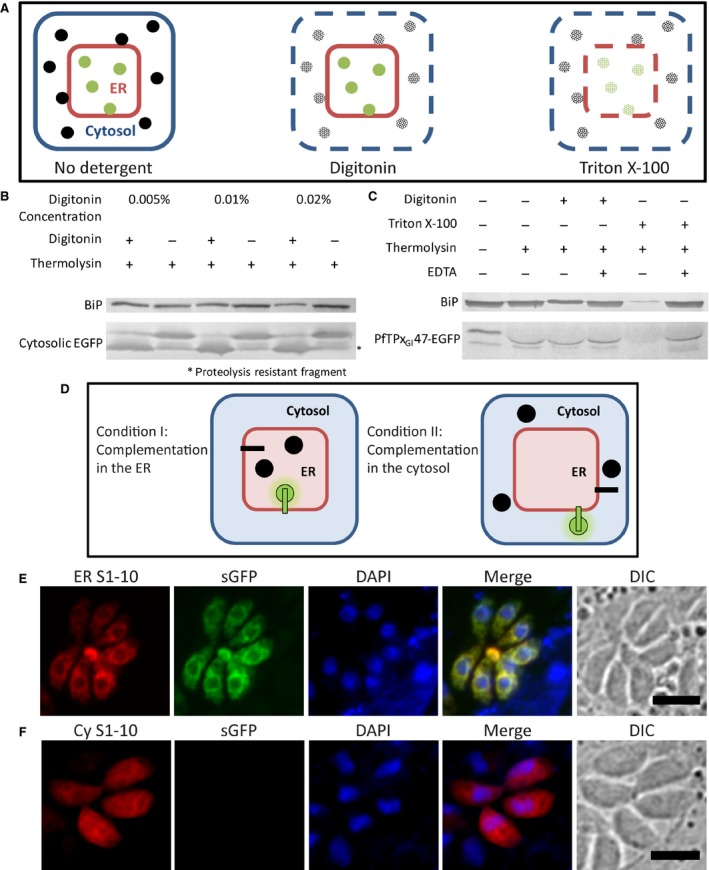
Assessment of PfTPx_Gl_47‐EGFP topology in the membrane. (A) Schematic representation of the protease protection assay. Digitonin permeabilization and protease treatment of parasites expressing (B) cytosolic EGFP for standardization and (C) PfTPx_Gl_47‐EGFP. (D) Schematic representation of the possible outcomes of split‐GFP complementation in the ER and the cytosol. Split‐GFP complementation in parasites expressing PfTPx_Gl_47‐S11, and either (E) ER luminal GFPS1‐10 or (F) cytosolic GFPS1‐10. Scale bar = 5 μm.

The first method employed to establish PfTPx_Gl_ topology was a protease protection assay. Here, we treated parasites with digitonin, a detergent that selectively permeabilizes the plasma membrane owing to its higher cholesterol content, but not the organellar membranes 44. These parasites were then incubated with the membrane‐impermeant protease thermolysin, which would diffuse across the permeabilized plasma membrane but not the intact ER membrane. Thus, if PfTPx_Gl_47‐EGFP faced the cytosol, it would be susceptible to proteolysis by thermolysin, and if it faced the ER lumen, it would be protected (Fig. 3A).

The concentration of digitonin that permeabilized the plasma membrane, but left the ER membrane intact, was standardized by testing for a lack of thermolysin‐induced degradation of the ER luminal protein BiP in parasites expressing cytosolic EGFP (Fig. 3B). At 0.005% digitonin, cytosolic EGFP was degraded, confirming plasma membrane permeabilization, while BiP was intact. This digitonin concentration was used to assay the orientation of PfTPx_Gl_47‐EGFP in the ER membrane.

In parasites treated with digitonin, both PfTPx_Gl_47‐EGFP and the ER luminal protein BiP remained intact in the presence of thermolysin, indicating that they were located in the same compartment and, therefore, that the C‐terminal EGFP domain was in the ER lumen (Fig. 3C). In contrast, in the presence of 1% Triton X‐100—a detergent that disrupted all membranes—both PfTPx_Gl_47‐EGFP and BiP were degraded, validating the observations in digitonin‐treated cells. EDTA was used to inhibit degradation in equivalent control reactions to confirm that the proteolysis was indeed a result of the specific enzymatic activity of thermolysin. The distortion of the PfTPx_Gl_47‐EGFP bands in the wells containing thermolysin might be a consequence of the large amounts of thermolysin running at the size of ~34 kDa. Incidentally, the enzymatic degradation of EGFP by thermolysin consistently resulted in a proteolysis‐resistant fragment (Fig. 3B), which has been reported elsewhere 45.

The second approach to confirm the PfTPx_Gl_47‐EGFP orientation was fluorescence complementation using split‐GFP 46. Split‐GFP is GFP that has been engineered as two separate parts—strands 1 to 10 (S1–10) of the barrel and strand 11 (S11)—that can each be expressed as fusions with two separate proteins. While neither S1–10 nor S11 fluoresces independently of the other, self‐assembly due to proximity *in vivo* results in fluorescence.

In this report, GFP S1–10 has been expressed either in the cytosol, free of targeting signals, or in the ER, tagged to P30 at the C terminus along with the ER retention/retrieval sequence HDEL. In order to assess the orientation of PfTPx_Gl_47 in the ER membrane, parasite lines expressing PfTPx_Gl_47 with a C‐terminal S11 tag were transiently transfected with both GFPS1–10 in the ER—ERS1–10—and GFPS1–10 in the cytosol—CyS1–10. If the C‐terminal S11 domain faced the ER lumen, split‐GFP complementation would occur in parasites expressing ERS1–10 within the ER (Condition I, Fig. 3D). Complementation in the parasites transfected with CyS1–10 would indicate that the C‐terminal S11 domain was located in the cytosol (Condition II, Fig. 3D).

The localizations of both ERS1–10 and CyS1–10 were confirmed by immunostaining with polyclonal anti‐GFP antibodies (Fig. 3E,F in red). The distinct perinuclear fluorescence staining pattern observed for ERS1–10 and the diffused fluorescence observed throughout the cell for CyS1–10 are patterns that are consistent with the expected ER and cytosolic localizations of these proteins. Green fluorescence was observed in the ER lumen only in parasite lines expressing PfTPx_Gl_47‐S11 that had been transfected with ERS1–10 (Fig. 3E), but not in the CyS1–10 transfectants (Fig. 3F). This indicated that complementation had occurred with ER luminal S1‐10 (Condition I, Fig. 3D), thereby confirming that the C terminus of the protein was positioned in the ER lumen.

Therefore, using two different approaches, the orientation of PfTPx_Gl_47‐EGFP was found to be such that the C‐terminal domain of the protein was in the ER lumen, while the N‐terminal transmembrane domain was anchored at the ER membrane.

### The two sections of the N terminus work in conjunction to ensure membrane anchorage and protein functionality

So far, this study has demonstrated that residues 1–21 of this N terminus are sufficient for targeting the protein to the endoplasmic reticulum, that the C terminus of this protein resides in the ER lumen, and that the N terminus of PfTPx_Gl_47‐EGFP is membrane anchored. If this is indeed the case, then it stands to reason that this transmembrane domain should be able to anchor a different downstream sequence to the ER membrane. Additionally, the role of residues 22–47, immediately preceding the glutathione peroxidase functional domain, remains to be assessed. In order to answer these questions, two chimeric constructs with a known *Toxoplasma* apicoplast‐targeted luminal protein, acyl carrier protein (ACP), were generated with EGFP tags. The first was a fusion protein of the signal peptide of ACP with residues 22–47 of PfTPx_Gl_ (ACP_SP_‐PfTPx_Gl_ΔS‐EGFP). The second was a fusion protein of residues 1–21 of PfTPx_Gl_ with the transit peptide of ACP (PfTPx_Gl_S‐ACP_TP_‐EGFP).

**Figure 4:**
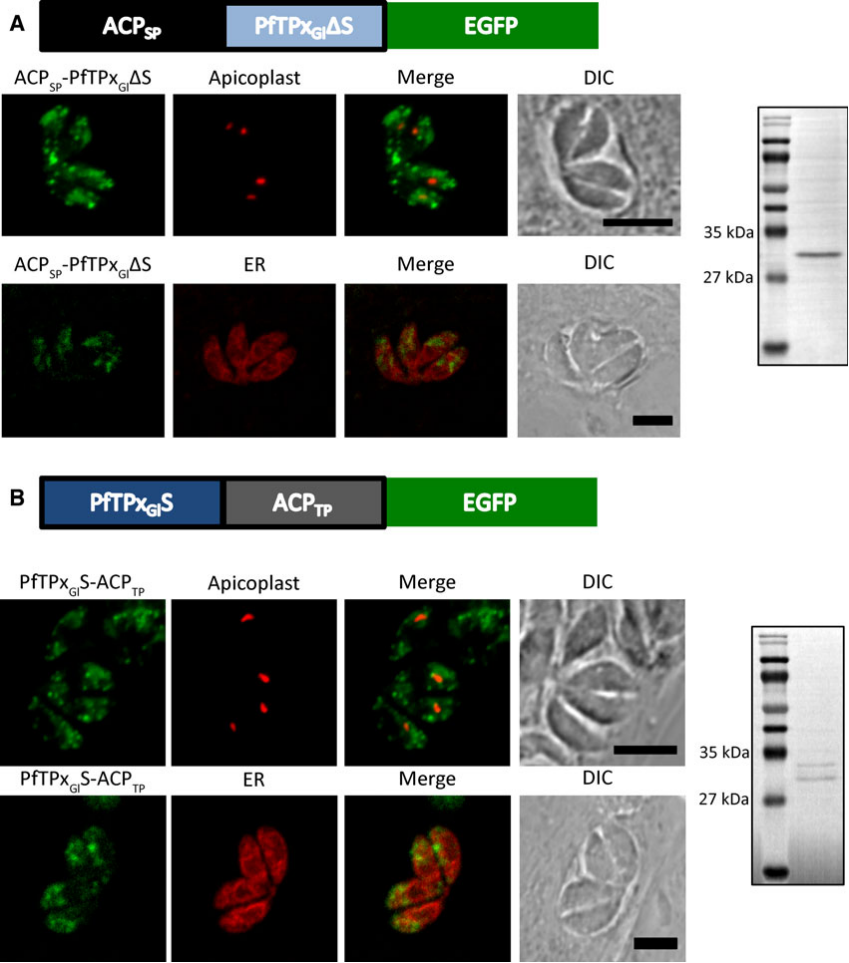
Mutational analysis of the PfTPx_Gl_ N terminus to study the signal sequence. Microscopic images of parasites expressing ACP and PfTPx_Gl_ chimeric constructs (A) ACP_SP_‐PfTPx_Gl_ΔS‐EGFP (green) and (B) PfTPx_Gl_S‐ACP_TP_‐EGFP (green). The marker for the apicoplast is FNR‐RFP (red), and for the ER is P30‐mCherry‐HDEL (red). Scale bar = 5 μm. Western blots of parasites expressing ACP_SP_‐PfTPx_Gl_ΔS‐EGFP and PfTPx_Gl_S‐ACP_TP_‐EGFP using anti‐GFP antibodies have been shown next to the respective constructs.

In parasites expressing ACP_SP_‐PfTPx_Gl_ΔS‐EGFP, western blots confirmed cleavage at the predicted site after the ACP signal peptide, resulting in a band that closely correlated with the expected size of 31.3 kDa (Fig. 4A). Upon imaging to assess whether residues 22–47 of PfTPx_Gl_ had a role in targeting within the endomembrane system, dispersed structures were observed throughout the parasite, suggesting that the protein had entered the post‐ER secretory system (Fig. 4A). This led us to conclude that the protein had escaped the ER lumen with the bulk flow. Indeed, in support of this, no colocalization was observed with the ER marker protein P30‐mCherry‐HDEL. Additionally, colocalization was not observed even with the apicoplast marker protein FNR‐RFP 47, indicating that in this context residues 22–47 possessed no apicoplast targeting information independently of residues 1–21.

In the case of PfTPx_Gl_S‐ACP_TP_‐EGFP, the protein was expected to remain anchored to the ER membrane. However, microscopy revealed that the protein had exited the ER and was located in the post‐ER secretory system (Fig. 4B), similar to the observation in parasites expressing ACP_SP_‐PfTPx_Gl_ΔS‐EGFP. This is supported by the lack of colocalization with the ER marker protein P30‐mCherry‐HDEL. Western blots revealed that this protein was indeed cleaved (Fig. 4B). While cleavage at the end of the predicted PfTPx_Gl_ signal sequence, at residue 21, would have resulted in a 35.4 kDa product, the size of the observed bands was indicative of cleavage further along the polypeptide chain, within the ACP transit peptide sequence, at more than one site. Additionally, the lack of colocalization with the apicoplast marker FNR‐RFP suggested that cleavage had taken place within the ACP transit peptide as it was no longer capable of mediating apicoplast localization. As this ACP transit peptide sequence is not conventionally cleaved, one might gather that in the absence of a strong cleavage consensus in the signal anchor of PfTPx_Gl_, alternative sites are preferred further downstream.

**Figure 5:**
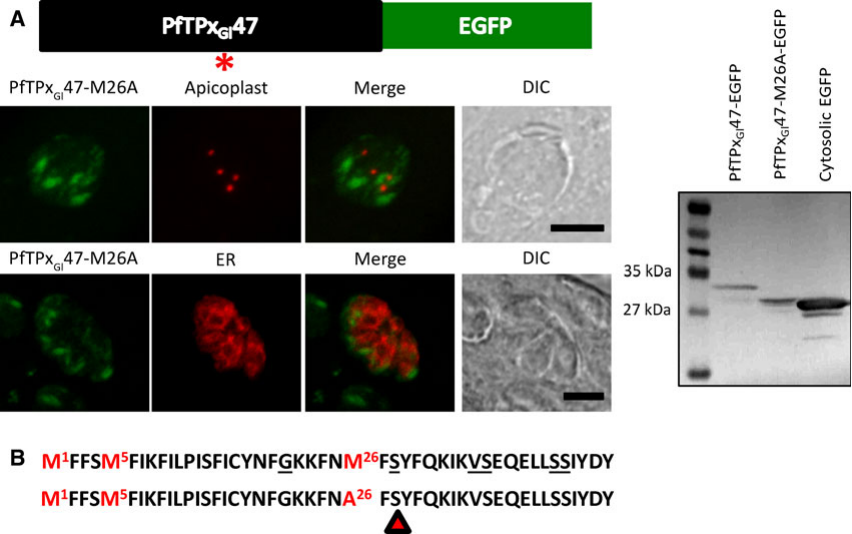
Mutational analysis of the PfTPx_Gl_ N terminus to study cleavage. (A) Microscopic images of PfTPx_Gl_47‐M26A‐EGFP (green). Apicoplast marker protein FNR‐RFP (red) and ER marker protein P30‐mCherry‐HDEL (red) are used for labeling the respective compartments. Scale bar = 5 μm. (A) Western blot of parasites expressing PfTPx_Gl_47‐M26A‐EGFP using anti‐GFP antibodies is shown next to the images. (B) The N‐terminal sequence of PfTPx_Gl_ displays the putative point mutations that can generate cleavage sites (underlined) and the expected site of cleavage in PfTPx_Gl_47‐M26A‐EGFP (red arrow).

This implies that generating a signal peptide cleavage site downstream of residue 21 of PfTPx_Gl_ would result in the cleavage of PfTPx_Gl_47‐EGFP. To test this hypothesis, the methionine at position 26 was mutated to an alanine, generating the construct PfTPx_Gl_47‐M26A‐EGFP. In theory, this should have generated a cleavage site immediately after the serine at position 28. Western blots of lysates of parasites expressing PfTPx_Gl_47‐M26A‐EGFP indicated that cleavage did actually take place at the predicted site, resulting in a band that closely matched the expected size of 30.4 kDa (Fig. 5A). Imaging of transfectants expressing this protein confirmed that the protein had exited the ER with the bulk flow and was located in the post‐ER secretory system. Notably, the protein did not colocalize with the ER marker protein P30‐mCherry‐HDEL, or with the apicoplast marker protein FNR‐RFP (Fig. 5A). From these data, it is evident that in the presence of a downstream cleavage site, the PfTPx_Gl_ signal anchor can serve as a canonical signal peptide.

### Transfection of the M26A mutant protein in *Plasmodium falciparum*

Once the signals in the PfTPx_Gl_ N terminus that were involved in the first step of the trafficking of this protein were characterized by mutational analysis in *T. gondii*, we decided to test one of the constructs in *P. falciparum*. The revelation that a single point mutation in the PfTPx_Gl_ N terminus could drive the protein to a different destination was the motivation for choosing to study the M26A mutant in *P. falciparum*. To ensure that these results were in agreement with the published literature 14, we also expressed the first 47 residues of native PfTPx_Gl_ fused to GFP in *P. falciparum*. Notably, in support of the premise of this report, the generation of these two transfectants and the acquisition of data took several months due to the host of complications discussed previously.

**Figure 6:**
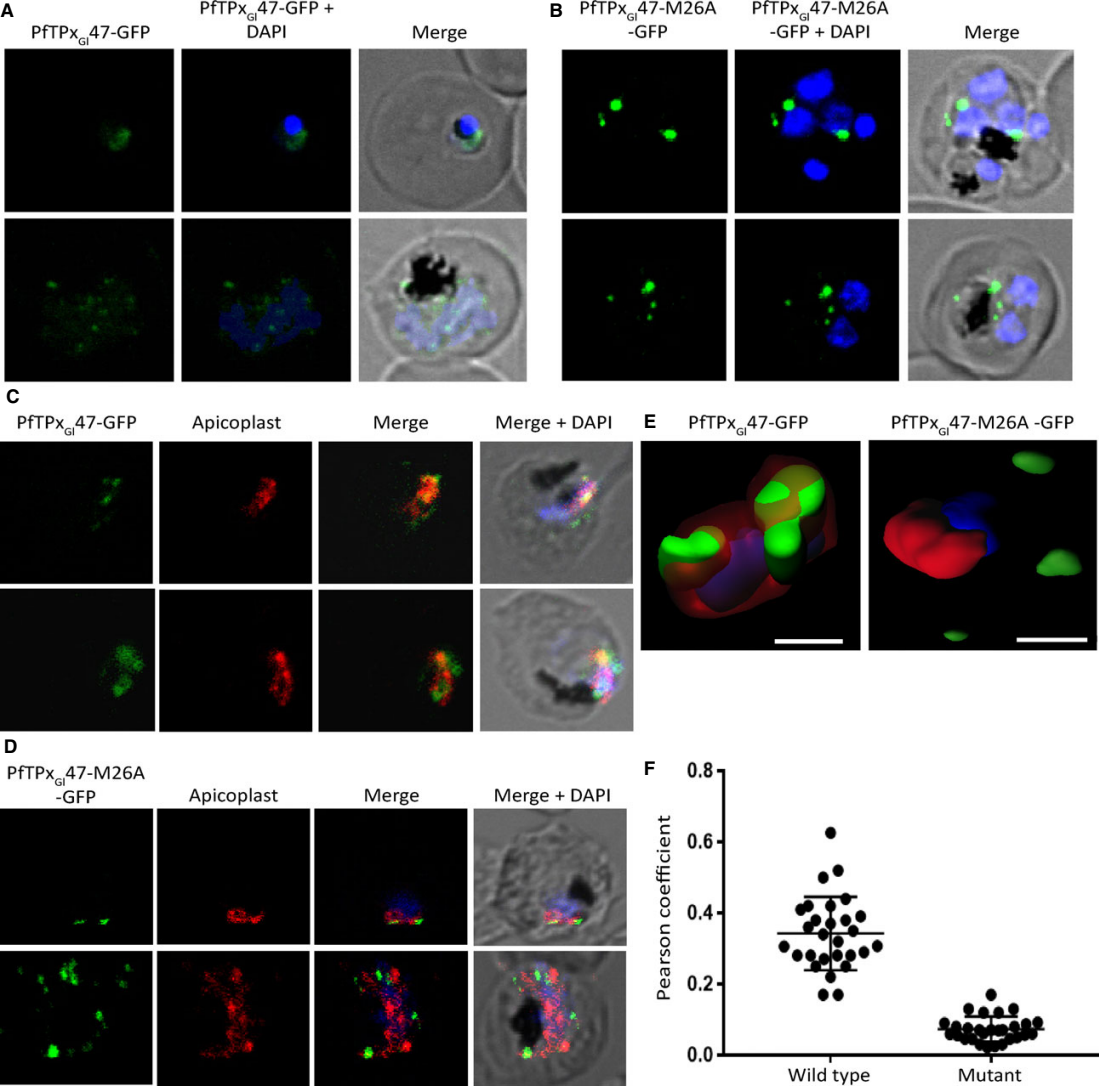
Expression of GFP fusions of native and mutated PfTPx_Gl_ N termini in *Plasmodium* transfectants. Immunofluorescence images of parasites expressing (A) PfTPx_Gl_47‐GFP (green) (B) PfTPx_Gl_47‐M26A‐GFP (green). Localization with the apicoplast marker protein PfClpP (red) is shown in parasites expressing (C) PfTPx_Gl_47‐GFP (green) and (D) PfTPx_Gl_47‐M26A‐GFP (green). (E) A 3D reconstruction using Z‐stack images of parasites expressing PfTPx_Gl_47‐GFP (green) or PfTPx_Gl_47‐M26A‐GFP (green) with apicoplast marker protein PfClp (red). Scale bar = 1 μm. (F) A dot plot of the Pearson's correlation coefficients for colocalization between the protein of interest and the apicoplast marker PfClpP in parasites expressing the N termini of the wild‐type (*n* = 28) and mutant (*n* = 28) proteins fused to GFP. The mean correlation coefficient, and therefore colocalization with PfClpP, is significantly higher for the wild‐type protein than for the mutant protein.

We observe that PfTPx_Gl_47‐GFP targeting in the parasite agrees very closely with the published data. In addition to faint, diffused fluorescence throughout the cell, indicative of cytosolic localization (Fig. 6A), the protein was also targeted to apicoplast in most but not all cells observed (Fig. 6C), as confirmed by colocalization with the apicoplast marker PfClpP 31. Three‐dimensional reconstructions using Z‐stacked images also showed colocalization of GFP and PfClpP staining (Fig. 6E, PfTPx_Gl_47‐GFP). The expression of PfTPx_Gl_47‐M26A‐GFP, on the other hand, resulted in targeting to punctate structures dispersed throughout the cell, with partial colocalization to the apicoplast (Fig. 6B,D) in a few cells. Here too, the three‐dimensional reconstructions were consistent with these observations (Fig. 6E, PfTPx_Gl_47‐M26A‐GFP).

The Pearson's correlation coefficient for the colocalization of the protein of interest with the apicoplast marker PfClpP was calculated for each parasite that was analyzed in the parasite line expressing the wild‐type protein (*n* = 28), as well as in the parasite line expressing the mutant protein (*n* = 28). A dot plot of the correlation coefficients in wild‐type and mutant parasite lines is displayed in Fig. 6F. A marked difference was observed between the mean correlation coefficient, representative of the extent of colocalization, of the wild‐type protein and that of the mutant protein with the apicoplast marker. There was no overlap in the data points, within one standard deviation of the mean, between the wild‐type and mutant protein data sets. Notably, the mean Pearson's correlation coefficient for the wild‐type protein was above 0.3, indicative of a moderate correlation 48, and therefore of the colocalization of the PfTPx_Gl_47‐GFP protein with the apicoplast marker 32. The mean correlation coefficient for the mutant protein, on the other hand, fell below the threshold of 0.1, which is considered the minimum value required for any data to qualify as having a small correlation 48. This suggested that the extent of colocalization of the mutant PfTPx_Gl_47‐M26A‐GFP protein with the apicoplast marker was negligible. From these results, it is evident that the M26A mutation alters localization in both *Toxoplasma* and *Plasmodium*, resulting in the respective proteins localizing to structures that are distinct from the apicoplast.

## Discussion

### *Toxoplasma gondii* as a heterologous system for studying *Plasmodium* biology

Admittedly, the generation of transfectants in *P. falciparum* makes the screening of multiple mutations and their effects on targeting time‐consuming as the low levels of DNA uptake result in longer recovery times under drug pressure 15, 16. In this study, *T. gondii*, a related apicomplexan, has been used to serve as an efficient preliminary screening system to identify a smaller number of relevant mutations whose influence on trafficking can then be verified in *P. falciparum*. While *Toxoplasma* has been used to express *Plasmodium* proteins for the generation of vaccines and the characterization of enzymes 49, 50, 52, to the best of our knowledge, this is the first report outlining the detailed dissection of the targeting signals of a *Plasmodium* protein in *Toxoplasma*. The similarity in the intracellular compartments between the two organisms coupled with the greater clarity in subcellular structure and increased efficiency of transfection makes *T. gondii* an intermediary in understanding certain aspects of protein trafficking in *P. falciparum* 1, 53, 54. This study highlights which of these aspects are suitable for study in *Toxoplasma*.

### The use of *Toxoplasma gondii* identifies the N‐terminal signal anchor in PfTPx_Gl_

Expressing the N terminus of PfTPx_Gl_ in *T. gondii* resulted in targeting to the endoplasmic reticulum. This is a fate differing from that in *P. falciparum*, where the N‐terminal 47 residues target the protein to the apicoplast and the cytosol. This difference in localization could be due to specific sequence requirements for apicoplast trafficking differing between the two parasites; for example, the lengths of predicted apicoplast targeting sequences are smaller in *P. falciparum* compared to *T. gondii* 55. The localization of this protein to the ER, however, was favorable as the ER is known to be the first step in trafficking proteins to the apicoplast. We decided therefore to exploit this phenotype to dissect the signals involved in ER uptake.

Mutational analysis of the N terminus of PfTPx_Gl_ defined a signal anchor sequence between residues 1–21—a signal sequence that targets the protein to the membrane of the endoplasmic reticulum. Membrane anchorage is consistent with results seen in *P. falciparum* organelles 13, and here, use of the heterologous system was able to define the N terminus of the protein as responsible for membrane anchorage and for targeting.

### Localization of the protein in the ER allows identification of membrane topology

Significantly, the C‐terminal domain of the protein was located in the ER lumen, indicating that this is most likely the topology retained upon reaching the apicoplast 42. The fortuitous localization of PfTPx_Gl_47‐EGFP to the ER membrane in *Toxoplasma* was extremely useful in determining the orientation of the protein using digitonin permeabilization that occurs in membranes enriched in cholesterol. The ER membrane contains less cholesterol than the plasma membrane allowing selective digitonin permeabilization while performing the protease protection assays. In contrast, protease protection assays to determine topology would have been challenging with the native protein localized to the apicoplast in *Plasmodium*. Here, as apicoplast membranes are enriched in cholesterol 56, digitonin would have permeabilized the organellar membrane as well as the plasma membrane, allowing the protease to digest PfTPx_Gl_ regardless of orientation.

Split‐GFP experiments to determine the orientation of the protein in the membrane also gave unambiguous results as markers for only the cytosol and ER lumen were required, and these markers were relatively simple to generate. The larger size and clarity in the ultrastructure of the *Toxoplasma* ER were key to obtaining clean results with split‐GFP. In contrast, to have done the same split‐GFP experiment in *Plasmodium* would have required a marker for the cytosol and another for the space between the outer membrane and the next membrane of the apicoplast. Only one apicoplast membrane protein has been definitively localized to this outer membrane, PfoTPT in *Plasmodium*. Terminal GFP tagging of this protein would not place the split‐GFP in the outermost compartment of the apicoplast because both the N termini and C termini of this protein reside in the cytosol 57. As little is known in both *Plasmodium* and *Toxoplasma* about the exact orientations of most apicoplast membrane proteins, or the specific membranes in which they reside, it would not have been feasible to conduct this experiment, while the protein was in the apicoplast membrane.

Our results, where the protein of interest was found in the ER, show that this localization provided an unexpected benefit for both the digitonin and split‐GFP strategies. We suggest that this approach of forcing a protein into the ER compartment, such as with an HDEL ER retention/retrieval signal, would enable the easier assessment of the membrane topology of Golgi‐dependent apicoplast proteins.

### Implications of the orientation of the PfTPx_Gl_

It has been implied that the orientation toward the ER lumen suggests a retention of the same orientation in the apicoplast membrane 42. Thus far, only proteins known to reside in the outermost membrane of the apicoplast have been reported to be devoid of cleavage/processing and a canonical bipartite targeting sequence, such as is observed for PfTPx_Gl_, while trafficking to this destination. If the ER orientation is retained, this would mean that in *P. falciparum*, the peroxidase domain of PfTPx_Gl_ should be located in the outermost compartment of the apicoplast, with the N terminus of the protein anchored to the outermost membrane. This orientation of the protein is significant as there are a large number of antioxidant proteins in the cytosol, but none reported so far in this compartment of the apicoplast in *Plasmodium* 14, 58. This would make PfTPx_Gl_ the first antioxidant enzyme reported that could handle oxidative stress in this niche. Additionally, this protein might serve as a bait in the outermost compartment of the apicoplast that could then be used to identify other proteins in this compartment.

### Membrane anchorage of the protein also depends on lack of a signal peptide cleavage site

As the protein is oriented toward the ER lumen, the residues downstream of the signal anchor are available to the signal peptidase. However, the protein is not cleaved, either in *Plasmodium* 10 or in *Toxoplasma* (this report). At a glance, the sequence of the PfTPx_Gl_ N terminus reveals enrichment in residues that are part of the signal peptide cleavage consensus 41, 59. Almost all of these residues, as highlighted in the figure, lie in close proximity to the signal anchor (Fig. 5B). This means that although not cleaved, there are many point mutations that can potentially result in any of these residues partaking in the formation of a functional cleavage site. When this hypothesis was tested by mutating the methionine at position 26 to an alanine, the protein was cleaved at the expected site (Fig. 5A). Given the ease of generation of cleavage sites, it might be speculated that the combination of residues in this stretch (residues 22–47) has been selected against cleavage in order to retain the membrane anchorage of the protein. Interestingly, while the M26A mutation resulted in a dramatic change (exit from the ER) in the localization in *Toxoplasma*, the change was more subtle was in *Plasmodium*. This difference also suggests that the two apicomplexan parasites do differ in certain aspects of the apicoplast trafficking machinery. Therefore, one must exercise discretion when using *Toxoplasma* as a heterologous system to study the trafficking of *Plasmodium* proteins.

The insights so far might lead one to question why such a long string of residues, with several potential cleavage sites, has been retained at all. The functional glutathione peroxidase domain of PfTPx_Gl_ begins at residue 43 (Conserved Domains Database). One might recall that in parasites expressing the PfTPx_Gl_ signal anchor (1–21) directly fused to EGFP in the absence of residues 22–47, native EGFP fluorescence was absent. However, anti‐GFP antibodies were effective at determining the localization of the protein. This indicated that the protein was targeted correctly but was misfolded, possibly due to steric hindrance from proximity to the membrane. From the data, it appears that residues 22–47 serve to space the functional domain from the membrane to ensure the proper folding of the protein.

## Conclusions

While *Toxoplasma* has previously been used to study *Plasmodium* vaccine candidates, enzymes 49, and even protein localization 9, 19, this is the first report outlining its utility in the dissection of some of the targeting signals of a *Plasmodium* protein.

This study successfully elucidates that PfTPx_Gl_ possesses an N‐terminal signal anchor at residues 1–21, with the C‐terminal domain in the organelle lumen. Residues 22–47 of the N terminus appear to serve the role of a spacer region lacking a cleavage site, so as to permit proper protein folding while reinforcing membrane anchorage. The use of *T. gondii*, in addition to expediting screening, has been particularly significant in the determination of the membrane topology of PfTPx_Gl_, an exercise that would have been difficult in its native context owing to the factors outlined. PfTPx_Gl_ is now in an emerging class of membrane‐anchored proteins that target to the *Plasmodium* apicoplast independently of the canonical bipartite sequence involved in the trafficking of apicoplast luminal proteins.

## Author contributions

AN designed and performed experiments, analyzed data, and wrote the paper. PM and VT performed experiments and analyzed data. PKR analyzed and critically interpreted the data; AM designed experiments and analyzed data. SP designed experiments, analyzed data, and wrote the paper.

## Conflict of interest

The authors declare no conflict of interest.

## References

[1] McFadden GI and Roos DS (1999) Apicomplexan plastids as drug targets. Trends Microbiol 7, 328–333. 10431206 10.1016/s0966-842x(99)01547-4

[2] Waller RF , Keeling PJ , Donald RG , Striepen B , Handman E , Lang‐Unnasch N , Cowman AF , Besra GS , Roos DS and McFadden GI (1998) Nuclear‐encoded proteins target to the plastid in *Toxoplasma gondii* and *Plasmodium falciparum* . Proc Natl Acad Sci USA 95, 12352–12357. 9770490 10.1073/pnas.95.21.12352 PMC22835

[3] Spork S , Hiss JA , Mandel K , Sommer M , Kooij TWA , Chu T , Schneider G , Maier UG and Przyborski JM (2009) An unusual ERAD‐like complex is targeted to the apicoplast of *Plasmodium falciparum* . Eukaryot Cell 8, 1134–1145. 19502583 10.1128/EC.00083-09 PMC2725561

[4] Kalanon M , Tonkin CJ and McFadden GI (2009) Characterization of two putative protein translocation components in the apicoplast of *Plasmodium falciparum* . Eukaryot Cell 8, 1146–1154. 19502580 10.1128/EC.00061-09 PMC2725556

[5] Sommer MS , Gould SB , Lehmann P , Gruber A , Przyborski JM and Maier U‐G (2007) Der1‐mediated preprotein import into the periplastid compartment of chromalveolates? Mol Biol Evol 24, 918–928. 17244602 10.1093/molbev/msm008

[6] Agrawal S and Striepen B (2010) More membranes, more proteins: complex protein import mechanisms into secondary plastids. Protist 161, 672–687. 21036664 10.1016/j.protis.2010.09.002 PMC3005297

[7] Lindner J , Meissner KA , Schettert I and Wrenger C (2013) Trafficked proteins‐druggable in *Plasmodium falciparum*? Int J Cell Biol 2013, 435981. 23710183 10.1155/2013/435981 PMC3655585

[8] Tonkin CJ , Struck NS , Mullin KA , Stimmler LM and McFadden GI (2006) Evidence for Golgi‐independent transport from the early secretory pathway to the plastid in malaria parasites. Mol Microbiol 61, 614–630. 16787449 10.1111/j.1365-2958.2006.05244.x

[9] Karnataki A , DeRocher A , Coppens I , Nash C , Feagin JE and Parsons M (2007) Cell cycle‐regulated vesicular trafficking of *Toxoplasma* APT1, a protein localized to multiple apicoplast membranes. Mol Microbiol 63, 1653–1668. 17367386 10.1111/j.1365-2958.2007.05619.x

[10] Chaudhari R , Narayan A and Patankar S (2012) A novel trafficking pathway in *Plasmodium falciparum* for the organellar localization of glutathione peroxidase‐like thioredoxin peroxidase. FEBS J 279, 3872–3888. 22889167 10.1111/j.1742-4658.2012.08746.x

[11] Heiny SR , Pautz S , Recker M and Przyborski JM (2014) Protein traffic to the *Plasmodium falciparum* apicoplast: evidence for a sorting branch point at the Golgi. Traffic 15, 1290–1304. 25264207 10.1111/tra.12226

[12] Lim L , Sayers CP , Goodman CD and McFadden GI (2016) Targeting of a transporter to the outer apicoplast membrane in the human malaria parasite *Plasmodium falciparum* . PLoS One 11, e0159603. 27442138 10.1371/journal.pone.0159603 PMC4956234

[13] Chaudhari R , Dey V , Narayan A , Sharma S and Patankar S (2017) Membrane and luminal proteins reach the apicoplast by different trafficking pathways in the malaria parasite *Plasmodium falciparum* . PeerJ 5, e3128. 28462015 10.7717/peerj.3128 PMC5410153

[14] Kehr S , Sturm N , Rahlfs S , Przyborski JM and Becker K (2010) Compartmentation of redox metabolism in malaria parasites. PLoS Pathog 6, e1001242. 21203490 10.1371/journal.ppat.1001242 PMC3009606

[15] Skinner‐Adams TS , Lawrie PM , Hawthorne PL , Gardiner DL and Trenholme KR (2003) Comparison of *Plasmodium falciparum* transfection methods. Malar J 2, 19. 12869208 10.1186/1475-2875-2-19 PMC166142

[16] Hasenkamp S , Russell KT and Horrocks P (2012) Comparison of the absolute and relative efficiencies of electroporation‐based transfection protocols for *Plasmodium falciparum* . Malar J 11, 210. 22720754 10.1186/1475-2875-11-210 PMC3407700

[17] Weiss LM and Kim K (2013) Toxoplasma gondii, 2nd edn. Elsevier Ltd, USA.

[18] Waller RF , Reed MB , Cowman AF and McFadden GI (2000) Protein trafficking to the plastid of *Plasmodium falciparum* is via the secretory pathway. EMBO J 19, 1794–1802. 10775264 10.1093/emboj/19.8.1794 PMC302007

[19] Jomaa H , Wiesner J , Sanderbrand S , Altincicek B , Weidemeyer C , Hintz M , Turbachova I , Eberl M , Zeidler J , Lichtenthaler HK *et al* (1999) Inhibitors of the nonmevalonate pathway of isoprenoid biosynthesis as antimalarial drugs. Science 285, 1573–1576. 10477522 10.1126/science.285.5433.1573

[20] van Dooren GG , Tomova C , Agrawal S , Humbel BM and Striepen B (2008) *Toxoplasma gondii* Tic20 is essential for apicoplast protein import. Proc Natl Acad Sci USA 105, 13574–13579. 18757752 10.1073/pnas.0803862105 PMC2533231

[21] Seeber F (1997) Consensus sequence of translational initiation sites from *Toxoplasma gondii* genes. Parasitol Res 83, 309–311. 9089733 10.1007/s004360050254

[22] Patakottu BR , Singh PK , Malhotra P , Chauhan VS and Patankar S (2012) *In vivo* analysis of translation initiation sites in *Plasmodium falciparum* . Mol Biol Rep 39, 2225–2232. 21643747 10.1007/s11033-011-0971-3

[23] Hager KM , Striepen B , Tilney LG and Roos DS (1999) The nuclear envelope serves as an intermediary between the ER and Golgi complex in the intracellular parasite *Toxoplasma gondii* . J Cell Sci 112 (Pt 16), 2631–2638. 10413671 10.1242/jcs.112.16.2631

[24] Bryksin AV and Matsumura I (2010) Overlap extension PCR cloning: a simple and reliable way to create recombinant plasmids. Biotechniques 48, 463–465. 20569222 10.2144/000113418 PMC3121328

[25] Sato S , Rangachari K and Wilson RJM (2003) Targeting GFP to the malarial mitochondrion. Mol Biochem Parasitol 130, 155–158. 12946854 10.1016/s0166-6851(03)00166-x

[26] Trager W and Jensen JB (1976) Human malaria parasites in continuous culture. Science 193, 673–675. 781840 10.1126/science.781840

[27] Lambros C and Vanderberg JP (1979) Synchronization of *Plasmodium falciparum* erythrocytic stages in culture. J Parasitol 65, 418–420. 383936

[28] Crabb BS , Rug M , Gilberger T‐W , Thompson JK , Triglia T , Maier AG and Cowman AF (2004) Transfection of the human malaria parasite *Plasmodium falciparum* . Methods Mol Biol Clifton NJ 270, 263–276. 10.1385/1-59259-793-9:263 15153633

[29] Schindelin J , Arganda‐Carreras I , Frise E *et al* (2012) Fiji: an open‐source platform for biological‐image analysis. Nat Methods 9 (7), 676‐682. 22743772 10.1038/nmeth.2019 PMC3855844

[30] Wickramarachchi T , Devi YS , Mohmmed A and Chauhan VS (2008) Identification and characterization of a novel *Plasmodium falciparum* merozoite apical protein involved in erythrocyte binding and invasion. PLoS ONE 3, e1732. 18320051 10.1371/journal.pone.0001732 PMC2253826

[31] Rathore S , Sinha D , Asad M , Böttcher T , Afrin F , Chauhan VS , Gupta D , Sieber SA and Mohmmed A (2010) A cyanobacterial serine protease of *Plasmodium falciparum* is targeted to the apicoplast and plays an important role in its growth and development. Mol Microbiol 77, 873–890. 20545854 10.1111/j.1365-2958.2010.07251.x

[32] Thakur V , Asad M , Jain S , Hossain ME , Gupta A , Kaur I , Rathore S , Ali S , Khan NJ and Mohmmed A (2015) Eps15 homology domain containing protein of *Plasmodium falciparum* (PfEHD) associates with endocytosis and vesicular trafficking towards neutral lipid storage site. Biochim Biophys Acta 1853, 2856–2869. 26284889 10.1016/j.bbamcr.2015.08.007

[33] Abas L and Luschnig C (2010) Maximum yields of microsomal‐type membranes from small amounts of plant material without requiring ultracentrifugation. Anal Biochem 401, 217–227. 20193653 10.1016/j.ab.2010.02.030 PMC3685806

[34] Bangs JD , Uyetake L , Brickman MJ , Balber AE and Boothroyd JC (1993) Molecular cloning and cellular localization of a BiP homologue in *Trypanosoma brucei*. Divergent ER retention signals in a lower eukaryote. J Cell Sci 105 (Pt 4), 1101–1113. 8227199 10.1242/jcs.105.4.1101

[35] Pino P , Foth BJ , Kwok L‐Y , Sheiner L , Schepers R , Soldati T and Soldati‐Favre D (2007) Dual targeting of antioxidant and metabolic enzymes to the mitochondrion and the apicoplast of *Toxoplasma gondii* . PLoS Pathog 3, e115. 17784785 10.1371/journal.ppat.0030115 PMC1959373

[36] Bendtsen JD , Nielsen H , von Heijne G and Brunak S (2004) Improved prediction of signal peptides: SignalP 3.0. J Mol Biol 340, 783–795. 15223320 10.1016/j.jmb.2004.05.028

[37] Cilingir G , Broschat SL and Lau AO (2012) ApicoAP: the first computational model for identifying apicoplast‐targeted proteins in multiple species of *Apicomplexa* . PLoS One 7, e36598. 22574192 10.1371/journal.pone.0036598 PMC3344922

[38] Kall L , Krogh A and Sonnhammer EL (2007) Advantages of combined transmembrane topology and signal peptide prediction‐the Phobius web server. Nucleic Acids Res 35, W429–W432. 17483518 10.1093/nar/gkm256 PMC1933244

[39] Fleige T , Fischer K , Ferguson DJP , Gross U and Bohne W (2007) Carbohydrate metabolism in the *Toxoplasma gondii* apicoplast: localization of three glycolytic isoenzymes, the single pyruvate dehydrogenase complex, and a plastid phosphate translocator. Eukaryot Cell 6, 984–996. 17449654 10.1128/EC.00061-07 PMC1951530

[40] Frankel MB and Knoll LJ (2009) The ins and outs of nuclear trafficking: unusual aspects in apicomplexan parasites. DNA Cell Biol 28, 277–284. 19348590 10.1089/dna.2009.0853 PMC2903460

[41] von Heijne G (1983) Patterns of amino acids near signal‐sequence cleavage sites. Eur J Biochem 133, 17–21. 6852022 10.1111/j.1432-1033.1983.tb07424.x

[42] Lim L , Kalanon M and McFadden GI (2009) New proteins in the apicoplast membranes: time to rethink apicoplast protein targeting. Trends Parasitol 25, 197–200. 19346163 10.1016/j.pt.2009.02.001

[43] Karsten V , Hegde RS , Sinai AP , Yang M and Joiner KA (2004) Transmembrane domain modulates sorting of membrane proteins in *Toxoplasma gondii* . J Biol Chem 279, 26052–26057. 15056659 10.1074/jbc.M400480200

[44] Fiskum G (1985) Intracellular levels and distribution of Ca2+ in digitonin‐permeabilized cells. Cell Calcium 6, 25–37. 4016893 10.1016/0143-4160(85)90032-6

[45] Leveque MF , Berry L , Cipriano MJ , Nguyen HM , Striepen B and Besteiro S (2015) Autophagy‐related protein ATG8 has a noncanonical function for apicoplast inheritance in *Toxoplasma gondii* . MBio 6, e01446‐15. 10.1128/mBio.01446-15 PMC4626856 26507233

[46] Cabantous S , Terwilliger TC and Waldo GS (2005) Protein tagging and detection with engineered self‐assembling fragments of green fluorescent protein. Nat Biotechnol 23, 102–107. 15580262 10.1038/nbt1044

[47] Striepen B , Crawford MJ , Shaw MK , Tilney LG , Seeber F and Roos DS (2000) The plastid of *Toxoplasma gondii* is divided by association with the centrosomes. J Cell Biol 151, 1423–1434. 11134072 10.1083/jcb.151.7.1423 PMC2150670

[48] Cohen J (1988) Statistical Power Analysis for the Behavioral Sciences, 2nd edn. Lawrence Erlbaum Associates, USA.

[49] Birkholtz LM , Blatch G , Coetzer TL , Hoppe HC , Human E , Morris EJ , Ngcete Z , Oldfield L , Roth R , Shonhai A *et al* (2008) Heterologous expression of plasmodial proteins for structural studies and functional annotation. Malar J 7, 197. 18828893 10.1186/1475-2875-7-197 PMC2567985

[50] Diaz CA , Allocco J , Powles MA , Yeung L , Donald RG , Anderson JW and Liberator PA (2006) Characterization of *Plasmodium falciparum* cGMP‐dependent protein kinase (PfPKG): antiparasitic activity of a PKG inhibitor. Mol Biochem Parasitol 146, 78–88. 16325279 10.1016/j.molbiopara.2005.10.020

[51] Di Cristina M , Ghouze F , Kocken CH , Naitza S , Cellini P , Soldati D , Thomas AW and Crisanti A (1999) Transformed *Toxoplasma gondii* tachyzoites expressing the circumsporozoite protein of *Plasmodium knowlesi* elicit a specific immune response in rhesus monkeys. Infect Immun 67, 1677–1682. 10085003 10.1128/iai.67.4.1677-1682.1999 PMC96513

[52] Haase S , Zimmermann D , Olshina MA , Wilkinson M , Fisher F , Tan YH , Stewart RJ , Tonkin CJ , Wong W , Kovar DR *et al* (2015) Disassembly activity of actin‐depolymerizing factor (ADF) is associated with distinct cellular processes in apicomplexan parasites. Mol Biol Cell 26, 3001–3012. 26157165 10.1091/mbc.E14-10-1427 PMC4551315

[53] Kim K and Weiss LM (2004) *Toxoplasma gondii*: the model apicomplexan. Int J Parasitol 34, 423–432. 15003501 10.1016/j.ijpara.2003.12.009 PMC3086386

[54] Roos DS , Crawford MJ , Donald RG , Fohl LM , Hager KM , Kissinger JC , Reynolds MG , Striepen B and Sullivan WJ Jr (1999) Transport and trafficking: *Toxoplasma* as a model for *Plasmodium* . Novartis Found Symp 226, 176‐95–8. 10.1002/9780470515730.ch13 10645546

[55] Seliverstov AV , Zverkov OA , Istomina SN , Pirogov SA and Kitsis PS (2015) Comparative analysis of apicoplast‐targeted protein extension lengths in apicomplexan parasites. Biomed Res Int 2015, 452958. 26114107 10.1155/2015/452958 PMC4465681

[56] Botte CY , Yamaryo‐Botte Y , Rupasinghe TW , Mullin KA , MacRae JI , Spurck TP , Kalanon M , Shears MJ , Coppel RL , Crellin PK *et al* (2013) Atypical lipid composition in the purified relict plastid (apicoplast) of malaria parasites. Proc Natl Acad Sci USA 110, 7506–7511. 23589867 10.1073/pnas.1301251110 PMC3645554

[57] Mullin KA , Lim L , Ralph SA , Spurck TP , Handman E and McFadden GI (2006) Membrane transporters in the relict plastid of malaria parasites. Proc Natl Acad Sci USA 103, 9572–9577. 16760253 10.1073/pnas.0602293103 PMC1480448

[58] Mohring F , Pretzel J , Jortzik E and Becker K (2014) The redox systems of *Plasmodium falciparum* and *Plasmodium vivax*: comparison, *in silico* analyses and inhibitor studies. Curr Med Chem 21, 1728–1756. 24304272 10.2174/0929867321666131201144612

[59] Nielsen H , Engelbrecht J , Brunak S and von Heijne G (1997) Identification of prokaryotic and eukaryotic signal peptides and prediction of their cleavage sites. Protein Eng 10, 1–6. 10.1093/protein/10.1.1 9051728

